# From the laboratory to the end-user: a primary packaging study for microneedle patches containing amoxicillin sodium

**DOI:** 10.1007/s13346-020-00883-5

**Published:** 2021-01-15

**Authors:** Emma McAlister, Mary-Carmel Kearney, E. Linzi Martin, Ryan F. Donnelly

**Affiliations:** Chair in Pharmaceutical Technology, School of Pharmacy, Queen’s University Belfast, 97 Lisburn Road, Belfast, BT9 7BL UK

**Keywords:** Microneedle patches, Hydrogel-forming microneedle arrays, Drug-containing reservoirs, Primary packaging, Amoxicillin sodium, Accelerated storage conditions

## Abstract

**Abstract:**

As microneedle (MN) patches progress towards commercialisation, there is a need to address issues surrounding their translation from the laboratory to the end-user. One important aspect of MN patches moving forward is appropriate primary packaging. This research focuses on MN patches containing amoxicillin (AMX) sodium for the potential treatment of neonatal sepsis in hot and humid countries. A MN patch consists of a hydrogel-forming MN array and a drug-containing reservoir. Improper primary packaging in hot and humid countries may result in degradation of active pharmaceutical ingredients, with the use of substandard medicines a major health concern. The research presented here, for the first time, seeks to investigate the integrity of MN patches in different primary packaging when stored under accelerated storage conditions, according to international guidelines. At pre-defined intervals, the performance of the MN patch was investigated. Major causes of drug instability are moisture and temperature. To avoid unnecessary degradation, suitable primary packaging was sought. After 168 days, the percentage of AMX sodium recovered from drug-containing reservoirs packaged in Protect™ 470 foil was 103.51 ± 7.03%. However, packaged in poly(ester) foil, the AMX sodium content decreased significantly (*p* = 0.0286), which is likely due to the degradation of AMX sodium by the imbibed moisture. Therefore, convincing evidence was provided as to the importance of investigating the stability of MN patches in primary packaging intended for MN-mediated transdermal delivery so that they are ‘fit for purpose’ when it reaches the end-user. Future work will include qualitative studies to assess MN patch usability.

**Graphical abstract:**

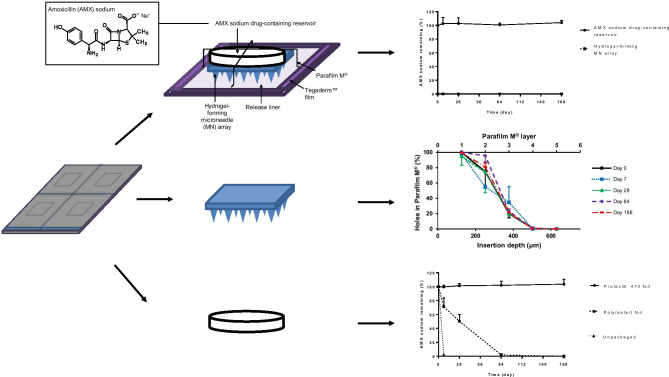

## Introduction

Microneedle (MN) arrays are minimally invasive devices that can painlessly bypass the *stratum corneum (SC)* and generate mechanical microchannels to facilitate drug delivery [1–3]. Since the first report of MN arrays in the literature [4], a variety of delivery strategies and material types have been described [5]. In this work, hydrogel-forming MN arrays are used. Comprised of chemically cross-linked hydrophilic polymer matrices [6], hydrogel-forming MN arrays are hard and sharp in the dry state. In this drug delivery strategy, the drug molecule to be delivered is not within the hydrogel-forming MN array but in a separate drug-containing reservoir. When both constituents are combined, it is referred to as a MN patch. Upon skin insertion, hydrogel-forming MN arrays rapidly imbibe skin interstitial fluid (ISF), swell and form continuous hydrogel pathways [6, 7]. The moisture from the swellable MN array comes in contact and subsequently triggers diffusion of the drug molecule from the attached drug-containing reservoir through the hydrogel matrix. This unique MN array design has its own advantages [8]. The degree of cross-linking in these MN arrays controls the drug release. Hydrogel-forming MN arrays are self-disabling, meaning that they cannot be reused, reducing the risk of needle stick injuries and infection transmission. Furthermore, the loading capacity is not limited by what can be loaded onto, or into the surface of the needles themselves. To date, an extensive number of studies have been published, showing the capabilities of hydrogel-forming MN arrays to transdermally deliver both small molecular weight drug molecules and macromolecules [8–12].

Moving towards large-scale manufacture and commercialisation, MN patches may well become one of the major pharmaceutical dosage forms and monitoring devices in the near future [13]. However, in order for new MN-based products/devices to release their undoubted potential and provide benefits for patient and industry, there is a need to address a number of factors surrounding the translation of MN patches from the laboratory setting to the end-user. Currently, studies in the literature have focused on the end-user themselves. Regarding patient application, we have shown that human volunteers can self-apply reproducibly hydrogel-forming MN arrays into their skin without an applicator [3, 14, 15]. In one study, this was completed by using a patient information leaflet and suitable counselling from a pharmacist [3]. In another study, we have shown how the incorporation of a simple pressure-indicating sensor film provided a level of assurance that hydrogel-forming MN array insertion had taken place [14]. Regarding patient acceptability, in one study, hydrogel-forming MN arrays were applied to human subjects over 65 years old [16]. By using focus groups, the overall consensus on the use of hydrogel-forming MN arrays was positive. Regarding patient safety, there is evidence to show that hydrogel-forming MN arrays exhibit no microbial growth, pose a very low risk to human health [3, 14–16] and cause no adverse side effects [17–19], even when repeatedly applied to the same human volunteers, every day for 5 days [20]. It was shown, for the first time, in human volunteers that repeat application and wear of hydrogel-forming MN arrays, prolonged skin reactions, or disruption of the skin barrier function did not occur and the normal balance of key systemic biomarkers over a fixed study period was not disrupted [20]. Although the commercial success of MN arrays will ultimately depend on the end-user, an important aspect that must be considered is if MN patches will be ‘fit for purpose’ when they reach the end-user. Accordingly, appropriate primary packaging must be sought for the storage, transport and distribution of MN patches.

Packaging is an integral part of a pharmaceutical product [21]. The main purpose of primary packaging is to ensure pharmaceutical product stability and protection against biological contamination, physical damage and external environmental conditions that can potentially affect the properties of the active pharmaceutical ingredient (API), such as light, moisture, oxygen and temperature [22, 23]. Commonly, there are three pharmaceutical packaging layers. Primary packaging is the packaging directly in contact and envelopes the pharmaceutical product, secondary packaging is for presentation/labelling purposes and tertiary packaging is added for bulk handling and shipping. MN technology will require specific consideration for primary packaging due to the principal function of these devices. For example, MN patches will not only need to maintain MN patch functionality but also ensure stability of the API for the lifetime of the product. Therefore, primary packaging used to store MN patches will need to maintain these characteristics over time.

Information on the stability of a new pharmaceutical product is an important part of the systematic approach to stability evaluation [24]. The purpose of stability testing is to provide evidence on how the quality of a pharmaceutical product varies with time under the influence of a variety of environmental factors such as temperature and relative humidity (RH) [24, 25]. To promote continuity and transferability worldwide for the process of stability testing, guidelines produced by the International Conference on Harmonisation (ICH) and the World Health Organisation (WHO) were adopted. The ICH guidelines divide the world into four climatic zones (with ICH climatic zone IV further subdivided into zones IVa and IVb). Based on temperature and RH, these climatic zones have been derived; due to the different environmental conditions, a drug product is most likely to be subjected to during their storage, transport and distribution within these regions [25]. This division ensures that the differences in temperature and RH in the varying regions of the world are considered in stability testing. ICH guidelines do not however address countries in ICH climatic zones III (hot and dry) and IV (hot and humid). Guidelines issued by the WHO specifically consider ICH climatic zones III and IV. Thus, both guidelines were adopted to cover the different environmental conditions, based on temperature and RH, in the world. Although these guidelines do not specify acceptance criteria, recommended parameters for stability testing are documented and the means of doing so [24, 25]. The recommended parameters include a container closure system (otherwise known as primary packaging), storage conditions, length of study, testing frequency and evaluation of the pharmaceutical product.

The stability of MN patches, consisting of hydrogel-forming MN arrays, enclosed in potential primary packaging and stored under accelerated storage conditions, according to international guidelines has never been investigated before. Considering other MN array delivery designs, in one study, dissolving MN arrays containing lidocaine hydrochloride were fabricated, packaged and stored under accelerated (40 ± 2 °C, 75 ± 5% RH) storage conditions for 84 days [26]. No significant changes occurred to the MN arrays after storage under these conditions [26]. In another study, dissolving MN arrays containing no drug were prepared and stored at 20 ± 2 °C, at three different RHs, namely 0, 43 and 75% RH for 21 days [27]. When stored at RHs of 43 and 75%, these dissolving MN arrays developed an increasingly adhesive nature and, hence, the MN array functionality was considerably affected. Furthermore, it is clear that in some studies discussed in the literature, stability testing of MN arrays in a controlled temperature and RH environment was evaluated, but the MN arrays were unpackaged [18, 28, 29].

We have recently described novel MN patches, consisting of hydrogel-forming MN arrays and directly compressed tablets (DCTs) containing amoxicillin (AMX) sodium [8]. Used as a low molecular weight model drug molecule in this work, AMX would have considerable utility in hot and humid countries if commercially developed using a MN patch for the treatment of neonatal sepsis. Approximately 420,000 new born babies die each year in hot and humid countries as a result of neonatal sepsis, despite the fact that there are effective treatments currently available [30]. Comparing MN patches containing AMX sodium with the conventional treatment of neonatal sepsis (oral AMX), apart from minimising the potential development of antibiotic resistance, MN patches do not require access to clean water for reconstitution purposes. Using this MN patch containing AMX sodium, we investigate, for the first time, the integrity of MN patches in different primary packaging when stored under accelerated storage conditions, according to international guidelines. Evaluation of the MN patch included firstly the MN array insertion properties; secondly, the AMX sodium-containing reservoir physical characteristics; and lastly, the recovery of AMX sodium from MN patches containing AMX sodium. By investigating, for the first time, the stability of MN patches in primary packaging according to international guidelines, which is a critical attribute for a marketed product, this study will add value to the MN field.

## Materials and methods

### Materials

AMX in the form of AMX sodium was purchased from Haihang Industry Co. (Jinan, Shandong Province, China). Poly(vinyl alcohol) (PVA) (MW 85–124 kDa), ammonium acetate, acetic acid, sodium hydroxide pellets, acetonitrile for high performance liquid chromatography (HPLC) and methanol Chromasol V^®^ for HPLC were all purchased from Sigma-Aldrich^®^ (St Louis, USA). Poly(vinyl pyrrolidone) (PVP) (MW 58 kDa), sold under the product brand name Plasdone™ K-29/32, was a gift from Ashland Pharmaceuticals (Kidderminster, UK). Citric acid was purchased from BHD Laboratory Supplies (Poole, UK). Trifluoroacetic acid (TFA) was purchased from Tokyo Chemical Industry UK Ltd. (Oxford, UK). Sodium starch glycolate (SSG), sold under the product brand name Primojel^®^, was obtained from DFE Pharma (Klever Strasse, Germany). All other chemicals used were of analytical reagent grade.

### Fabrication of hydrogel-forming MN arrays

**Fig. 1:**
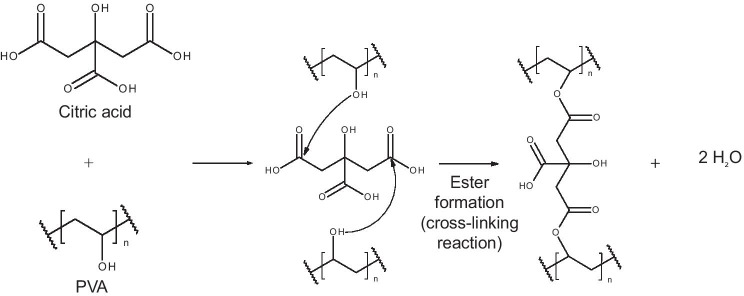
Schematic representation of the chemical structures and proposed chemical reaction that takes place during the cross-linking process between citric acid and PVA

Hydrogel-forming MN arrays were prepared as previously described [8]. Containing no drug themselves, PVA, PVP and citric acid were mixed to produce an aqueous blend consisting of a final composition of 15% w/w PVA, 10% w/w PVP and 1.5% citric acid. The aqueous blend (0.5 g) was dispensed into pre-fabricated silicone laser-engineered MN array moulds [7, 31]. These moulds consisted of 361 (19 × 19) needles, perpendicular to the base and of conical shape and height 600 µm, with base width 300 µm and needle interspacing 150 µm on a 0.49 cm^2^ area. The moulds, containing the aqueous blend, were centrifuged at 3500 repetitions per minute (rpm) for 15 min and then dried at room temperature for 48 h. After drying, the MN arrays were removed from the mould, with sidewalls subsequently removed using a pair scissors. The MN arrays were then placed on a glass petri dish and heated at 130 °C for 3 h. Heating the MN arrays facilitated the cross-linking esterification reaction between the carboxylic acid groups of the citric acid and the hydroxyl functional groups of the PVA [32]. A schematic representation of the proposed cross-linking chemical reaction between citric acid and PVA is shown in Fig. 1.

### Preparation of AMX sodium DCTs

To be used in conjunction with hydrogel-forming MN arrays, AMX sodium DCTs, as drug-containing reservoirs, were prepared. Previously optimised and characterised [8], AMX sodium DCTs were prepared using 95% w/w AMX sodium and 5% w/w SSG. In a pestle and mortar, both components were mixed in their dry state. Approximately 200 mg of the resulting formulation was placed into a dye (diameter 13 mm) which was placed into a Specac^®^ Atlas manual hydraulic press (Specac^®^ Ltd., Kent, UK), and pressure of 1 t was applied for 20 s. An average of 190 mg AMX sodium was contained in each AMX sodium DCT.

### Study protocol

Stability testing was conducted in accordance to ICH and WHO guidelines [24, 25]. For the study protocol, the parameters determined for stability testing included a container closure system (otherwise known as primary packaging), storage conditions, length of study, testing frequency, and evaluation of the pharmaceutical product. In this study, there were three cohorts. Two different primary packaging were investigated, namely Protect™ 470 MIL-PRF-131 K Class 1 (Protect™ 470) foil (Protective Packaging Corporation, Texas, USA) and heat-sealable poly(ester) foil (Transparent Film Products Ltd, Bangor, Northern Ireland). Both primary packaging was separated into four (*n* = 4) individual compartments via heat sealing. This was to facilitate more replicate testing in each primary packaging and to avoid unnecessary migration of AMX sodium (or other excipients) within the same primary packaging. In the primary packaging groups, when all contents were placed inside the packaging, it was sealed (with a double seal) using an Impulse Hand Sealer™ (Pac Seal, Etten-Leur, The Netherlands). The seal ability of the foil following heat sealing in all primary packaging was visually checked. With this, prior to exposure to accelerated storage conditions, seal integrity studies were performed by immersion of the primary packaging into 1% v/v amaranth solution. After 2 min, the sealed foils were removed and inspected for internal discolouration after opening. The control cohort was unpackaged.

**Fig. 2:**
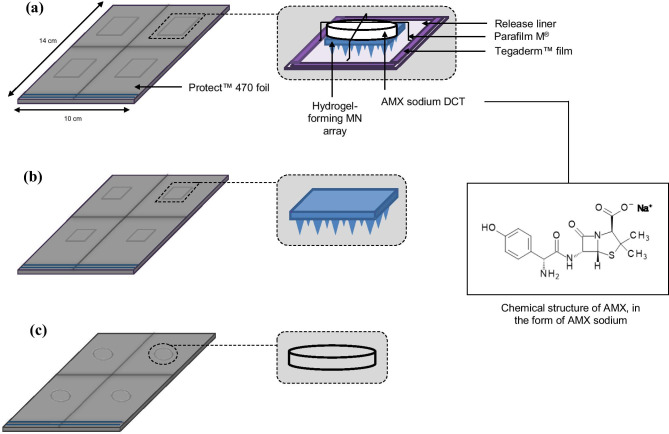
Schematic representation of the three groups packaged in Protect™ 470 foil; (**a**) MN patches, consisting of hydrogel-forming MN arrays and AMX sodium DCTs. MN patch constituents were fixed to one another using release liner, Parafilm M^®^ and Tegaderm™ film; (**b**) hydrogel-forming MN arrays and (**c**) AMX sodium DCTs

Constituents, hydrogel-forming MN arrays and AMX sodium DCTs formed three groups. The first group was MN patches which consists of hydrogel-forming MN arrays and AMX sodium DCTs. AMX sodium DCTs were placed on top of hydrogel-forming MN arrays and held in place using release liner, FL2000 PET 75 (Rexam Release B.V., Apeldoorn, The Netherlands), Parafilm M^®^ laboratory film (Bemis Company Inc, Soignies, Belgium) and Tegaderm™ film (3 M, St Paul, Minnesota, USA). Release liner was used as a base layer (25 × 25 mm). Strips of Parafilm M^®^ were placed over the MN patch and strips of Tegaderm™ film were used to secure the Parafilm M^®^ strips to the release liner. These materials were selected because of their non-toxic properties. The second group was hydrogel-forming MN arrays, and the third group was AMX sodium DCTs. A schematic representation of the three groups packaged in Protect™ 470 foil is illustrated in Fig. 2. When all cohorts were exposed to accelerated storage conditions, the orientation of the MN patch was noted [24]. For consistency, all hydrogel-forming MN arrays were placed into the constant climate chamber with the needles facing downwards.

In accordance to ICH and WHO guidelines [24, 25], stability testing was conducted by storing all three cohorts in a Binder KBF constant climate chamber (Binder GmbH, Tuttlingen, Germany) at accelerated (40 ± 2 °C, 75 ± 5% RH) storage conditions for 168 days. At pre-defined intervals (7, 28, 84 and 168 days), samples were removed from the constant climate chamber. It is important to highlight that samples removed for testing did not return to the constant climate chamber. Thus, a sufficient number of groups within each cohort were prepared to achieve suitable testing at each time interval. In all cases, all groups were visually inspected. The parameters assessed following removal of groups from accelerated storage conditions included percentage of AMX sodium remaining from both MN patch constituents (separately), insertion properties of hydrogel-forming MN arrays, physical characterisation and percentage of AMX sodium remaining from AMX sodium DCTs.

### Percentage of AMX sodium remaining from MN patches consisting of hydrogel-forming MN arrays and AMX sodium DCTs

The constituents of the MN patch were initially separated. Absorbed/adsorbed AMX sodium was washed off by individually placing hydrogel-forming MN arrays into 5 ml ammonium acetate buffer (pH 5.76) for 5 min at 37 ± 1 °C. The percentage of AMX sodium washed off the hydrogel-forming MN array was determined using reversed-phase (RP)-HPLC. In a parallel test, AMX sodium DCTs were individually disintegrated in ammonium acetate buffer (pH 5.76), stirred at 600 rpm and temperature maintained at 37 ± 1 °C. Following disintegration, the percentage of AMX sodium remaining was analysed and quantified using RP-HPLC.

### Insertion properties of hydrogel-forming MN arrays

The insertion properties of hydrogel-forming MN arrays were investigated using a previously described and validated test method [33]. Briefly, in this test method, Parafilm M^®^ was used as a model membrane. One sheet of Parafilm M^®^ was carefully folded, such that it formed eight layers, approximately 1 mm thick. This was then placed onto a piece of dental wax (Kemdent Works, Swindon, UK) wrapped in aluminium foil (Wrap Film Systems Ltd, Telford, England) before being placed onto the stage of a TA.XT.Plus Texture Analyser (Stable Micro Systems Ltd, Godalming, UK). Hydrogel-forming MN arrays were attached to the moveable probe of the Texture Analyser using double-sided sticky tape and applied perpendicularly into the eight-layer film of Parafilm M^®^. In compression mode, the probe of the Texture Analyser was programmed to lower at a test speed of 1.19 mm/s and at a force of 32 N for 30 s. After 30 s, the probe moved upward at a test speed of 10.0 mm/s. Following removal of the hydrogel-forming MN array from the Parafilm M^®^, the number of holes in each Parafilm M^®^ layer were counted using a Leica EZ4 D digital light microscope (Leica Microsystems, Milton Keynes, UK). To aid the visualisation of holes in each Parafilm M^®^ layer when viewed under the digital light microscope, two 82 mm Fotodiox circular polariser filters (Fotodiox, Inc., Gurnee, USA) were used. Each layer Parafilm M^® ^was placed between these two polariser filters. Using Eq. 1, the percentage of holes in each layer was calculated. From this, an approximate insertion depth was also ascertained.1, the percentage of holes in each layer was calculated. From this, an approximate insertion depth was also ascertained.

1 $${\mathrm{Holes in Parafilm M}}^{\mathrm{\circledR }} (\mathrm{\%})=\frac{\mathrm{number of holes observed}}{\mathrm{number of holes expected}}*100$$

The height of individual needles on hydrogel-forming MN arrays (*n* = 16) was measured before and after the insertion study using the digital light microscope. The percentage change in height of the needles on hydrogel-forming MN arrays following insertion into Parafilm M^®^ was calculated using Eq. 2, where *H*_BI_ is the height of an individual needle on the hydrogel-forming MN array before insertion into the eight layer film of Parafilm M^®^ and *H*_AI_ is the height after insertion.

2 $$\mathrm{\%\;change\;in\;needle\;height }=\left(\frac{{H}_{\mathrm{BI}}- {H}_{\mathrm{AI}}}{{H}_{\mathrm{BI}}}\right)* 100$$

### Physical characterisation and percentage of AMX sodium remaining from AMX sodium DCTs

Physical characterisation studies were performed on AMX sodium DCTs. Initially, the mass of each AMX sodium DCT was recorded. Tablet dimensions, diameter and thickness of each AMX sodium DCT were then measured using digital callipers (Jade Products Rugby Limited, Warwickshire, UK). In each case, mean values were determined. The break force of AMX sodium DCTs was determined using a fracturability test method, as previously described [8]. Briefly, the Texture Analyser was set up such that two aluminium blocks, secured to the Texture Analyser stage, were set at a distance of 6.5 mm apart and a cuboidal aluminium probe (length 56 mm) was connected to the Texture Analyser. Each AMX sodium DCT was placed horizontally onto the aluminium blocks and the blunt probe connected to the Texture Analyser was centrally located over the AMX sodium DCT. In compression mode, the probe of the Texture Analyser was programmed to lower at a test speed of 1 mm/s and travel to a total distance of 6 mm upon contact with the AMX sodium DCT. Using the capabilities of the Texture Analyser software, the maximum force to cause breakage of the AMX sodium DCT along its central axis was determined from force-distance plots produced by the Exponent Software on the Texture Analyser. From this, a mean value at each time interval was determined. Tablet hardness was assessed using a test method outlined in the British Pharmacopoeia [34]. In this test method, an AMX sodium DCT was placed on the tablet stage between the jaws of a Dr. Schleuniger Pharmatron tablet hardness tester (Copley Scientific, Nottingham, UK) and compressed. The minimum, maximum and mean force to fracture the AMX sodium DCT at each pre-defined time interval, expressed in Newtons (N), was recorded.

In another set of AMX sodium DCT samples, stored under accelerated storage conditions, individual AMX sodium DCTs were placed in 20 ml ammonium acetate buffer (pH 5.76). Individual AMX sodium DCTs were stirred at 600 rpm and temperature maintained at 37 ± 1 °C. Following disintegration of AMX sodium DCTs, the percentage of AMX sodium remaining was determined. Samples were diluted appropriately, filtered and analysed using RP-HPLC.

### Pharmaceutical analysis

A RP-HPLC method was developed with isocratic elution to analyse AMX sodium in ammonium acetate buffer (pH 5.76) following the studies described above. The method, previously described [8], was used for this work. In brief, the method was achieved on an Agilent 1200 series system, and Chemstation^®^ computer software B.02.01 was used for chromatogram analysis. The column was a SphereClone™ C_18_ ODS(1) (80 Å pore size, 150 mm length × 4.6 mm internal diameter; 5 µm particle size) (Phenomenex, Cheshire, UK) with the temperature of the column maintained at 15 °C. The mobile phase consisted of 95:05% v/v 0.02 M ammonium acetate buffer (0.1% v/v TFA, adjusted to pH 5.0) and acetonitrile/methanol (50:50). Mobile phase buffer was filtered and degassed by sonication prior to use for 1 h. The flow rate was 1 ml/min, and the injection volume was 20 µl. UV detection was set at 228 nm. The sample run time was 10 min, and the retention time for AMX sodium was 3.3 min. Standard samples of AMX sodium (2.5–125 µg/ml) were prepared in ammonium acetate buffer (pH 5.76).

The RP-HPLC method developed for the detection and quantification of AMX sodium in ammonium acetate buffer (pH 5.76) was validated according to ICH guidelines [35]. The parameters assessed during method validation were specificity, linearity, range, accuracy, precision, limit of detection (LoD) and limit of quantification (LoQ). All the calibration plots were subsequently collated to generate one representative calibration curve. Least squares linear regression analysis and correlation analysis were performed. The LoD and LoQ were determined using the standard deviation (S.D.) of the response and slope of the calibration curve, as described in the ICH guidelines [35]. AMX sodium validation parameters are documented in Table 1.

**Table 1:** AMX sodium validation parameters for RP-HPLC method, AMX sodium in ammonium acetate buffer (pH 5.76) (means ± S.D., *n* = 9)

Analytical method	Range (µg/ml)	Slope	*y*-intercept	r^2^	LoD (µg/ml)	LoQ (µg/ml)
AMX sodium in ammonium acetate buffer (pH 5.76)	2.5–125	8.50	0.86	1.0000	0.53	1.60

Where necessary, stability samples were diluted in ammonium acetate buffer (pH 5.76) prior to RP-HPLC analysis. AMX sodium concentrations (${\left[\mathrm{AMX sodium}\right]}_{\mathrm{sample}})$ were calculated using Eq. 3. In this equation, ${\mathrm{AUC}}_{\mathrm{sample}}$ is the area under the curve (AUC) representing AMX sodium, ${\left[\mathrm{AMX sodium}\right]}_{\mathrm{standard}}$ is the external standard concentration of AMX sodium and ${\mathrm{AUC}}_{\mathrm{standard}}$ is the AUC of the external standard of AMX sodium. Stability samples were compared with an external AMX sodium standard concentration of 50 µg/ml.

3 $${\left[\mathrm{AMX sodium}\right]}_{\mathrm{sample}}= \frac{{\mathrm{AUC}}_{\mathrm{sample}}* {\left[\mathrm{AMX sodium}\right]}_{\mathrm{standard}}}{{\mathrm{AUC}}_{\mathrm{standard}}}*\begin{array}{c}\mathrm{dilution}\\ \mathrm{factor}\end{array}$$

In this work, the recovery (%) of AMX sodium was calculated in some cases. This was determined, using Eq. 4.

4 $$\mathrm{Recovery }\left(\mathrm{\%}\right)= \frac{\mathrm{Amount of AMX sodium obtained}}{\mathrm{Amount of AMX sodium expected}}*100$$

### Statistical analysis

GraphPad Prism^®^ version 5.0 (GraphPad Software Inc., San Diego, California) was used to perform statistical analysis. Where appropriate, Mann-Whitney *U* tests were performed for comparison of two unpaired groups when *n* ˂ 5. The Wilcoxan matched-pairs signed rank test was used for comparison of two paired groups when *n* < 5. All data was expressed as means ± S.D.. Statistical significance was denoted when *p* ˂ 0.05 in all cases.

## Results

### Stability testing under accelerated storage conditions

**Fig. 3:**
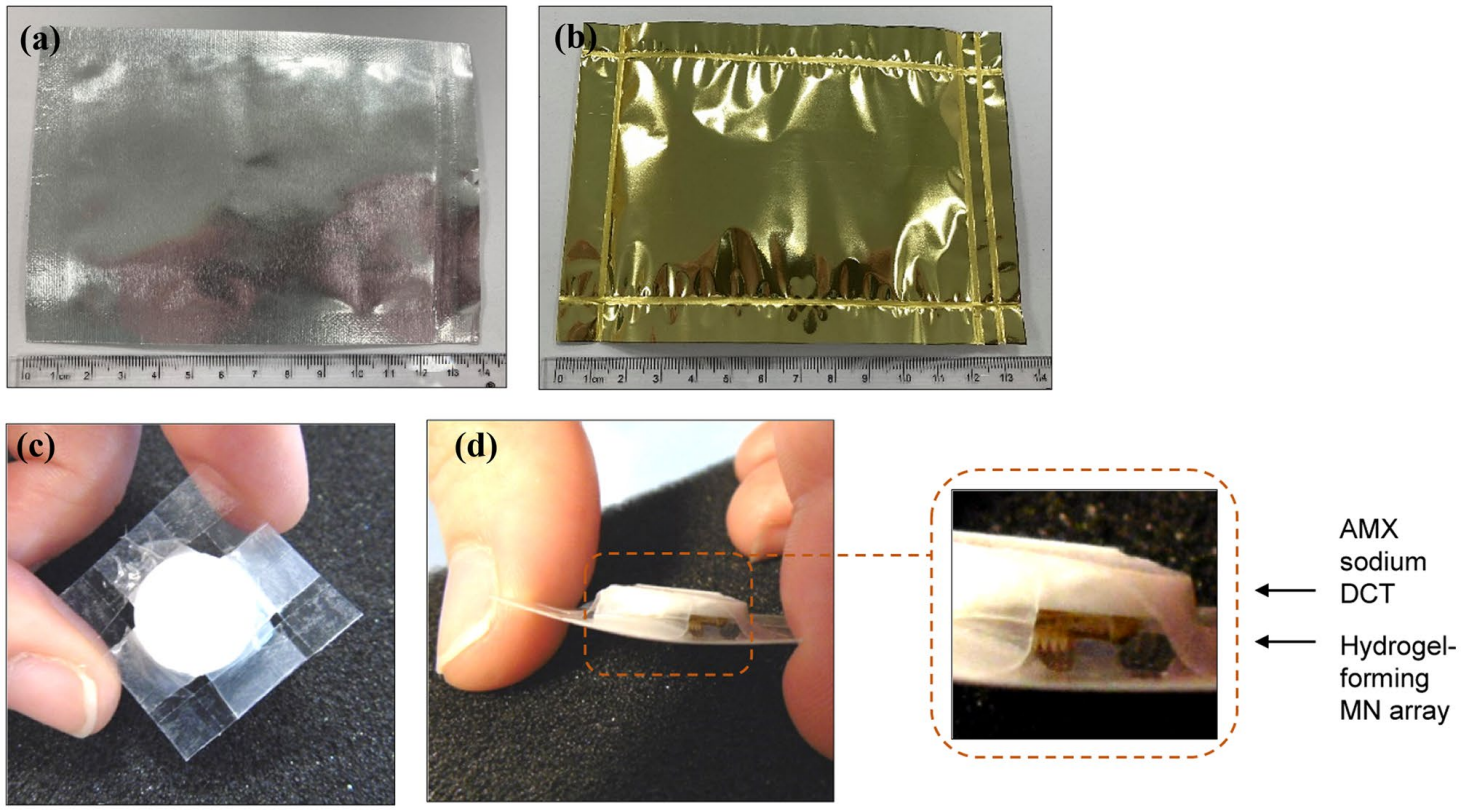
(**a–b**) Digital images of primary packaging used during stability testing under accelerated storage conditions; (**a**) Protect™ 470 foil and (**b**) poly(ester) foil. (**c–d**) Digital images of MN patches; (**c**) plan view and (**d**) lateral view with expanded image showing more clearly the MN patch consisting of hydrogel-forming MN array and AMX sodium DCT

The stability testing of MN patches, hydrogel-forming MN arrays and AMX sodium DCTs was evaluated under accelerated storage conditions. There were three cohorts; one cohort packaged in Protect™ 470 foil (Fig. 3a), one cohort packaged in poly(ester) foil (Fig. 3b) and one cohort as the control which was unpackaged. For those unpackaged, 6-well Nunclon™ delta surface disposables for cell culture (Thermo Fisher Scientific, Kamstrup, Denmark), discarding the lids, were used for storage purposes in the constant climate chamber. In completing seal integrity studies, no internal discolouration was observed in both foils. Within each cohort, there were three groups; MN patches (Fig. 3c, d), hydrogel-forming MN arrays and AMX sodium DCTs. The constituents of the MN patch (a hydrogel-forming MN array and an AMX sodium DCT) were secured to one another using release liner, Parafilm M^®^ and Tegaderm™ film.

For the experimental test period, in the primary packaging groups, there was no visible change in either of the primary packaging during stability testing under accelerated storage conditions. In other words, there was no loss of primary packaging functionality.

At each time interval, samples were removed from the accelerated storage conditions and were initially visually examined. All results were compared with the reference source (day 0). The results are presented in Tables 2, 3, and 4. Cohort one, packaged in Protect™ 470 foil is illustrated in Table 2 and Cohort two, packaged in poly(ester) foil is illustrated in Table 3. The unpackaged cohort is illustrated in Table 4.

**Table 2:**
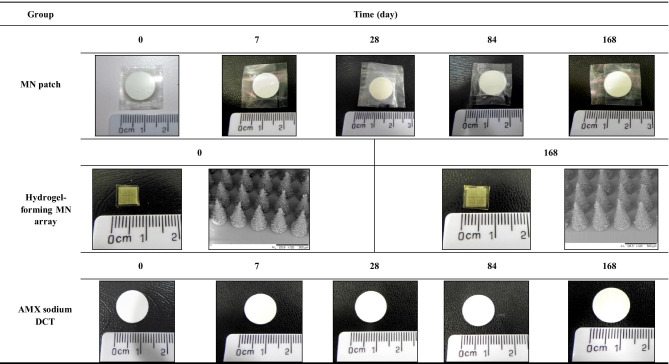
Digital images of MN patches, hydrogel-forming MN arrays, and AMX sodium DCTs following removal from Protect™ 470 foil after being stored under accelerated storage conditions over 168 days

**Table 3:**
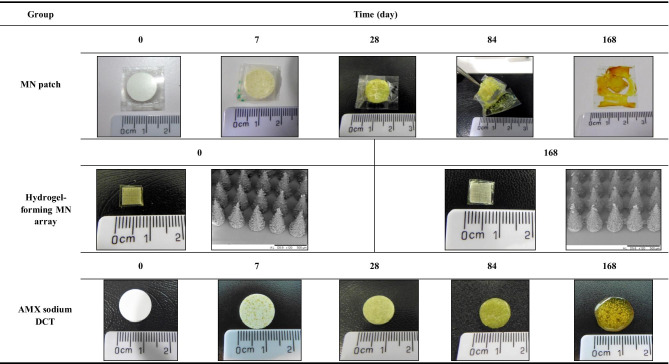
Digital images of MN patches, hydrogel-forming MN arrays, and AMX sodium DCTs following removal from poly(ester) foil after being stored under accelerated storage conditions over 168 days

**Table 4:**
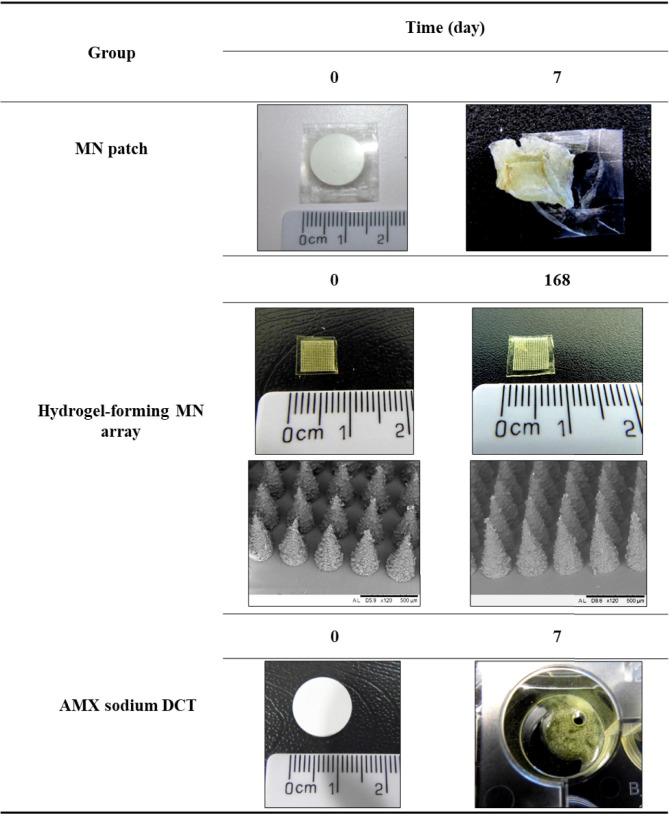
Digital images of unpackaged MN patches, hydrogel-forming MN arrays, and AMX sodium DCTs after being stored under accelerated storage conditions over 168 days

No visible differences were observed in MN patches packaged in Protect™ 470 foil and stored under accelerated conditions for 168 days (Table 2). AMX sodium DCTs within the MN patch remained white in colour after 168 days. With regards to hydrogel-forming MN arrays, plan SEM images showed no visible differences between day 0 and day 168 (day 0 and day 168 only shown). With regard to individual AMX sodium DCTs, AMX sodium DCTs also remained white in colour after 168 days stored under accelerated storage conditions.

Visible differences were clearly observed for MN patches packaged in poly(ester) foil and stored under accelerated storage conditions over 168 days (Table 3). At day 7, yellow speaks were observed on the surface of the MN patch constituent, AMX sodium DCTs. At day 28, distinct yellow AMX sodium DCTs were observed, and at day 84, the constituents of the MN patch were not easily separated from one another. At day 168, the AMX sodium DCT was distorted in shape and brown in colour. A ‘sulphur-like’ smell was also noted upon opening of the poly(ester) foils at day 28. With regards to individual hydrogel-forming MN arrays, similar to those packaged in Protect™ 470 foil, no visible differences were observed after 168 days, stored under accelerated storage conditions (day 0 and day 168 only shown). With regard to AMX sodium DCTs individually packaged in poly(ester) foil, considerable changes were observed. These changes were visibly similar to the AMX sodium DCTs within the MN patches packaged in poly(ester) foil as previously discussed. At day 7, yellow specks were observed on the surface of AMX sodium DCTs. At days 28 and 84, distinct yellow AMX sodium DCTs were observed with the yellow colour increasing in intensity, the longer the AMX sodium DCTs were stored under accelerated storage conditions. At day 168, brown AMX sodium DCTs were observed. Interestingly, at day 168, AMX sodium DCTs remained intact but the AMX sodium DCTs were distorted in shape. Similar to the MN patches packaged in poly(ester) foil, a ‘sulphur-like’ smell was noted upon opening of the poly(ester) foils at day 28 containing AMX sodium DCTs.

Visible differences were observed for MN patches unpackaged and stored under accelerated storage conditions (Table 4). At day 7, both constituents of the MN patch were not easily separated. With regard to individual hydrogel-forming MN arrays, as previously seen in the primary packaging cohorts, no visible differences were observed after 168 days of storage under accelerated storage conditions (day 0 and day 168 only shown).With regards to individual AMX sodium DCTs, at day 7, the AMX sodium DCTs were yellow in colour and had lost their circular shape. They also could not be removed from the Nunc™ multi dishes. As a result, MN patches and AMX sodium DCTs unpackaged beyond day 7 were no longer analysed.

#### Percentage of AMX sodium remaining from MN patches consisting of hydrogel-forming MN arrays and AMX sodium DCTs

**Fig. 4:**
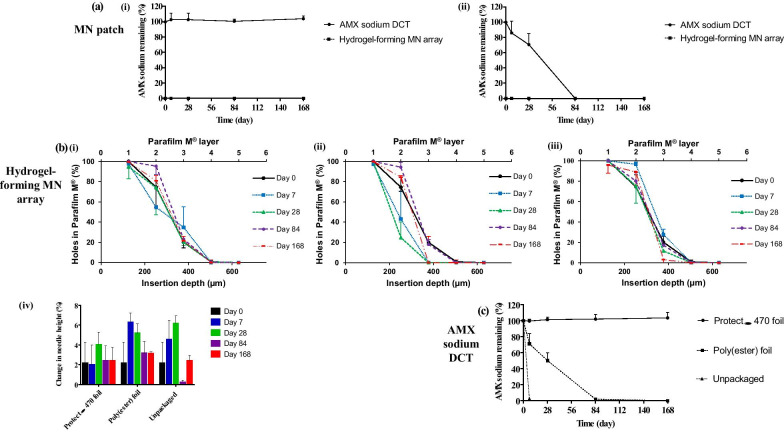
(**a**) Percentage of AMX sodium remaining in MN patch constituents, hydrogel-forming MN arrays, and AMX sodium DCTs, packaged in (**i**) Protect™ 470 foil and (**ii**) poly(ester) foil (means ± S.D., *n* ≥ 3). (**b**) Number of holes created in each Parafilm M^®^ layer expressed as a percentage to the number of holes expected and approximate insertion depth following insertion of hydrogel-forming MN arrays into Parafilm M^®^ after packaged in (**i**) Protect™ 470 foil, (**ii**) poly(ester) foil, or (**iii**) unpackaged (means ± S.D., *n* ≥ 3). (**iv**) Percentage change in needle height determined via the mean needle height pre- and post-insertion into Parafilm M^®^ (means ± S.D., *n* ≥ 3) and (**c**) percentage of AMX sodium remaining from AMX sodium DCTs packaged in Protect™ 470 foil, poly(ester) foil, and unpackaged (means ± S.D., *n* ≥ 3)

At pre-defined intervals, MN patches were removed from accelerated storage conditions and the MN patch was separated into its constituents. The percentage of AMX sodium remaining in each constituent, separately, was assessed. The results of the MN patches packaged in Protect™ 470 foil and poly(ester) foil are presented in Fig. 4(a)(i) and (a)(ii), respectively. The initial percentage of AMX sodium in AMX sodium DCTs at day 0 was 99.87 ± 1.35%. Regarding the control cohort, both constituents of the MN patch could not be separated, thus the migration potential of AMX sodium could not be analysed.

At day 168, no AMX sodium was quantified in hydrogel-forming MN arrays packaged in Protect™ 470 foil. It was also noted that the percentage of AMX sodium from AMX sodium DCTs remained consistent throughout the test period with 103.94 ± 3.37% of AMX sodium remaining in the AMX sodium DCTs at day 168. This resulted in a non-significant difference in the percentage of AMX sodium remaining between day 0 and day 168 when packaged in Protect™ 470 foil (*p* = 0.1143).

At day 168, no AMX sodium was quantified in hydrogel-forming MN arrays packaged in poly(ester) foil. However, it was noted that there was a considerable reduction in the percentage of AMX sodium in AMX sodium DCTs packed in poly(ester) foil over the test period. The percentage of AMX sodium remaining in the AMX sodium DCTs was 85.81 ± 15.32% at day 7 and 70.50 ± 14.31% at day 28. Statistically, there was no significant difference between the percentage of AMX sodium remaining at day 7, compared with day 0 (*p* = 0.0571). However, there was a significant difference between the percentage of AMX sodium remaining at day 28, compared with day 0 (*p* = 0.0286). Between day 28 and day 84, the percentage of AMX sodium considerably decreased. At day 84, the percentage of AMX sodium remaining was 0.15 ± 0.06% and 0.13 ± 0.05% at day 168.

#### Insertion properties of hydrogel-forming MN arrays

At pre-defined intervals, the insertion capabilities of hydrogel-forming MN arrays into Parafilm M^®^ were investigated. In all cases, it was noticed that hydrogel-forming MN arrays remained intact without any breakage of needles after removal from Parafilm M^®^. The results of hydrogel-forming MN arrays packaged in Protect™ 470 foil are presented in Fig. 4(b)(i). Packaged in Protect™ 470 foil, the results at each time interval were similar to the reference (day 0). At all-time intervals, hydrogel-forming MN arrays could insert into three layers of Parafilm M^®^, with 21.42 ± 6.80% holes created in layer three of Parafilm M^®^ from MN arrays at day 168. The mean thickness of a Parafilm M^®^ layer is 126 ± 7 µm; this suggests that hydrogel-forming MN arrays at day 168 inserted up to 378 µm of the total 600 µm, which is approximately > 50% of the total needle height. Figure 4(b)(ii) depicts the insertion capabilities of hydrogel-forming MN arrays in the cohort packaged in poly(ester) foil. Despite no visible differences in hydrogel-forming MN arrays over 168 days, at day 7 and thereafter, MN arrays packaged in poly(ester) foil were incapable of inserting into three layers of Parafilm M^®^. For example, at day 7, 43.01 ± 27.09% holes were created in layer two of Parafilm M^®^ with no visible holes created in layer three. This suggests that hydrogel-forming MN arrays at day 7 inserted up to 252 µm of the total 600 µm, which is 42% of the total needle height. Figure 4 (b)(iii) shows the insertion capabilities of hydrogel-forming MN arrays in the unpackaged cohort after stability testing up to 168 days. Similar to the cohort packaged in poly(ester) foil, despite no differences visible in hydrogel-forming MN arrays over 168 days, at day 28 and thereafter, MN arrays unpackaged were incapable of inserting considerably into three layers of Parafilm M^®^. For example, at day 28, 11.45 ± 0.80% holes were created in layer three of Parafilm M^®^. This suggests that hydrogel-forming MN arrays at day 28 inserted up to 252 µm of the total 600 µm, which is 42% of the total needle height.

The percentage change in needle height of hydrogel-forming MN arrays packaged in Protect™ 470 foil, packaged in poly(ester) foil or unpackaged was determined pre- and post-insertion into Parafilm M^®^, as presented in Fig. 4(b)(iv). Regarding hydrogel-forming MN arrays packaged in Protect™ 470 foil, needle height had decreased the most by 3.11 ± 4.92% for MN arrays at day 28. With this, at each time interval, there was no significant difference, between the mean needle height pre- and post-insertion into Parafilm M^®^ for hydrogel-forming MN arrays packaged in Protect™ 470 foil at each time interval (*p* = 0.7500 at day 7, *p* = 0.1250 at day 28, *p* = 0.6250 at day 84 and *p* = 0.2500 at day 168). For hydrogel-forming MN arrays packaged in poly(ester) foil, needle height had decreased the most by 5.62 ± 6.94% at day 7. Also, there was no significant difference, between the mean needle height pre- and post-insertion into Parafilm M^®^for hydrogel-forming MN arrays packaged in poly(ester) foil at each time interval (*p* = 0.1250 at day 7, *p* = 0.2500 at day 28, *p* = 0.0947 at day 84 and *p* = 0.1250 at day 168). For hydrogel-forming MN arrays in the unpackaged cohort, needle height had decreased the most by 6.73 ± 5.59% for hydrogel-forming MN arrays at day 28. Similar to both primary packaging cohorts, there was no significant difference, between the mean needle height pre- and post-insertion into Parafilm M^®^ for hydrogel-forming MN arrays unpackaged at each time interval (*p* = 0.2500 at day 7, *p* = 0.2500 at day 28, *p* = 1.0000 at day 84 and *p* = 0.1250 at day 168).

#### Physical characterisation and percentage of AMX sodium remaining from AMX sodium DCTs

At pre-defined intervals, AMX sodium DCTs were physically characterised. The results of AMX sodium DCTs packaged in Protect™ 470 foil and poly(ester) foil are presented in Table 5, respectively. AMX sodium DCTs unpackaged could not be physically characterised, because they could not be easily handled, as previously illustrated in Table 4. Packaged in Protect™ 470 foil, AMX sodium DCTs demonstrated uniform mass and physical dimensions (diameter and thickness). Fracturability (break force) and hardness tests were measured. Comparing results at day 0 and day 168, AMX sodium DCTs at day 168 were not significantly different in terms of the force to break (*p* = 0.8571) or force to crush (*p* = 0.6286) when packaged in Protect™ 470 foil. However, packaged in poly(ester) foil, comparing results at day 0 and day 168, AMX sodium DCTs at day 168 significantly increased in mass (*p* = 0.0286), diameter (*p* = 0.0294) and thickness (*p* = 0.0286). In terms of fracturability and hardness comparisons at day 0 and day 168, AMX sodium DCTs at day 168 required a significantly higher force to break (*p* = 0.0286) and were significantly harder to crush (*p* = 0.0498).

**Table 5:** Physical characterisation of AMX sodium DCTs packaged in (a) Protect™ 470 foil and (b) poly(ester) foil (means ± S.D., *n* ≥ 3)

				**Time (day)**		
(a)						
Parameter		0	7	28	84	168
Mass (mg)		194.68 ± 2.62	193.75 ± 2.27	200.45 ± 1.52	201.28 ± 0.26	200.53 ± 1.36
Diameter (mm)		13.02 ± 0.01	13.04 ± 0.01	13.03 ± 0.05	13.04 ± 0.02	13.03 ± 0.01
Thickness (mm)		1.30 ± 0.03	1.32 ± 0.03	1.29 ± 0.04	1.32 ± 0.03	1.33 ± 0.02
Break force (*N*)		2.77 ± 0.55	2.97 ± 0.48	2.79 ± 0.45	2.71 ± 0.33	2.77 ± 0.30
Hardness (*N*)	Minimum	18	29	25	23	21
	Maximum	30	35	34	31	31
	Mean	24.00 ± 6.00	31.67 ± 3.06	28.75 ± 4.11	27.00 ± 4.08	26.50 ± 4.20

				**Time (day)**		
(b)						
Parameter		0	7	28	84	168
Mass (mg)		194.68 ± 2.62	193.13 ± 3.95	212.78 ± 6.45	233.68 ± 3.31	249.53 ± 3.43
Diameter (mm)		13.02 ± 0.01	13.15 ± 0.02	13.24 ± 0.09	13.32 ± 0.05	14.02 ± 0.80
Thickness (mm)		1.30 ± 0.03	1.37 ± 0.02	212.78 ± 6.45	1.39 ± 0.01	1.75 ± 0.37
Break force (*N*)		2.77 ± 0.55	2.60 ± 0.35	1.38 ± 0.00	3.43 ± 0.58	9.90 ± 2.07
Hardness (*N*)	Minimum	18	19	29	31	39
	Maximum	30	34	41	50	43
	Mean	24.00 ± 6.00	25.75 ± 6.24	34.25 ± 5.12	43.33 ± 10.69	40.50 ± 1.91

The percentage of AMX sodium remaining from AMX sodium DCTs in each cohort was determined and is presented in Fig. 4(c). Packaged in Protect™ 470 foil, the percentage of AMX sodium remaining in AMX sodium DCTs did not substantially reduce. For example, at day 168, 103.51 ± 7.03% of AMX sodium was remaining. Statistically, there was no significant difference between the percentage of AMX sodium remaining at day 168 compared with day 0 (*p* = 0.6286). However, a considerable reduction in the percentage of AMX sodium occurred in AMX sodium DCTs packaged in poly(ester) foil. The percentage of AMX sodium remaining from AMX sodium DCTs was 71.19 ± 12.62% at day 7 and 50.25 ± 9.69% at day 28. Statistically, there was a significant difference between the percentage of AMX sodium remaining at day 7 compared with day 0 (*p* = 0.0286). Between day 28 and day 84, the percentage of AMX sodium considerably decreased. At day 84, the percentage of AMX sodium remaining from AMX sodium DCTs was 1.92 ± 0.92% and 0.03 ± 0.02% at day 168. Furthermore, it was noted that a similar trend in the percentage of AMX sodium remaining in AMX sodium DCTs was reported in Fig. 4(a)(i) and (a)(ii), where the percentage of AMX sodium remaining from AMX sodium DCTs in the MN patches was calculated. When the unpackaged MN patches were sampled at day 7, it was found that only 2.21 ± 0.21% of AMX sodium remained.

## Discussion

The emergence of antibiotic resistance is currently one of the greatest global health challenges [36]. As compared with oral delivery of AMX, transdermal drug delivery is a favourable alternative route of administration. The primary consideration is the fact that it avoids first pass metabolism. By circumventing the gastrointestinal tract, not only is gastrointestinal degradation prevented but the potential development of antibiotic resistance is minimised, thus extending the lifespan of existing antibiotics. Many drug molecules, however, do not possess the specific physiochemical properties necessary to passively transverse the main skin barrier, the *SC.* MN arrays can therefore be used to enhance transdermal drug delivery. We have recently shown, as proof of concept evidence that AMX sodium can be delivered at therapeutically relevant concentrations in vivo using novel MN patches [8]. The effects of primary packaging for the storage, transport and distribution of MN patches was investigated in this work.

The use of substandard or degraded medicines represents a huge threat to health because they can inadvertently lead to health care failures, spread of a disease within a community and subsequently death [37, 38]. A collaborative study was conducted to evaluate the quality of AMX-clavulanic acid preparations from different manufacturers in hot and humid counties [37]. Unintentional degradation of the API occurred due to the improper primary packaging, storage and handling of drug products. The major causes of drug instability are moisture and temperature [23]. Regarding this work, these environmental factors could considerably affect the delivery capabilities of MN patches containing AMX sodium. Following an ingress of moisture, hydrogel-forming MN arrays would soften and therefore hinder their ability to insert successfully into the skin. AMX sodium is inherently unstable, as hydrolysis occurs, due to the presence of a labile, highly strained β-lactam ring [39]. AMX degrades to initially form AMX penicilloic acid, AMX penilloic acid and diketopiperazine AMX, with several further degradation products also identified [40–42]. Thus, the degradation of AMX due to hydrolysis is complex. The hygroscopic nature of AMX sodium and temperature sensitivity has been documented [43]. With this, the moisture sorption properties of SSG has been reported, which in turn can influence the superdisintegrant’s effectiveness [44]. As a result, these environmental factors could cause non-functional and unstable MN patches to be formed.

Primary packaging selection is considered to be one of the most important decisions during formulation development of a pharmaceutical product as its main purpose is to maintain drug stability and provide a protective barrier to external environmental conditions [37, 45]. To avoid unnecessary degradation, moisture sorption or temperature issues, suitable primary packaging, in terms of moisture barrier function, temperature resistance and good heat sealant properties were sought. In this work, one foil claiming moisture and heat barrier properties was purchased, namely Protect™ 470 foil (Protective Packaging Corporation, 2018). Another foil, namely poly(ester) foil, was already available in the laboratory setting. Poly(ester) foil has previously demonstrated its success during stability testing for bioadhesive films [45]. With this in mind, we wanted to investigate if this poly(ester) foil was suitable for these MN patches in this study. The control cohort consisted of unpackaged groups.

Stability testing of a new pharmaceutical product is an important regulatory requirement. To ensure the correct conditions were implemented, guidelines on stability testing from ICH and WHO were adopted. This is because stability testing in accordance with international guidelines provides documented evidence and a level of assurance that the MN patch is suitable for it intended purpose when it reaches the end-user. For stability testing, it is common for a stability protocol to be created and followed [46, 47]. This is a written document that describes the key components that must be considered and implemented during stability testing. Key components include primary packaging, storage conditions, length of study, testing frequency and evaluation of the pharmaceutical product. This is by no means an exhaustive list, but the main components considered in this study. ICH and WHO guidelines recommend that storage conditions and length of study should be sufficient to cover storage, shipment and subsequent use with regard to the climatic conditions in which the product is intended to be marketed [24, 25]. As advised by the guidelines, all new pharmaceutical products are recommended to be tested under accelerated storage conditions for a minimum of 6 months, irrespective of the climate conditions prevalent in the target countries [24, 25]. By definition, accelerated storage conditions use exaggerated storage conditions to evaluate the effect of short-term excursions outside the label storage conditions, such as during shipping. As these MN patches are intended for use in hot and humid countries, these storage conditions may relate to the potentially extreme harsh environmental conditions the primary packaging (containing MN patches) could be exposed to. Therefore, these storage conditions were followed in this work. Stability testing under accelerated storage conditions recommends a minimum of three time intervals, including the initial and final time points from the 6-month study [24, 25]. In this study, the testing frequency was increased to five-time intervals to ensure the integrity of the MN patch was carefully monitored.

The performance of the MN patch at each pre-defined time interval was evaluated. Performance parameters included hydrogel-forming MN array functionality, in terms of insertion properties, AMX sodium DCT physical characteristics and recovery of AMX sodium. These key parameters were assessed as they were considered to be critical quality attributes for the success of this novel drug delivery system. Initial work began via visual examination. Confirmation of physical degradation of AMX sodium was provided via visible discolouration of AMX sodium DCTs (in the MN patch and individually) when packaged in poly(ester) foil and unpackaged. Reports of physical instability of AMX can be found in the literature [48]. The change of a pharmaceutical products physical appearance can dictate appropriate primary packaging [49]. However, it is important to note that visible examination is not a quantitative method and therefore it is almost impossible to use to accurately predict the effects of temperature and moisture.

Initial quantitative studies were completed on the MN patches. This work demonstrated that AMX sodium from AMX sodium DCTs did not migrate into its corresponding hydrogel-forming MN array during stability testing when packaged in Protect™ 470 foil. Migration of AMX sodium during storage could considerably change the delivery capabilities of these MN patches. To explain this, if diffusion of AMX sodium through the hydrogel-forming MN arrays initiated during storage, AMX sodium would be available more readily upon MN array insertion/fluid uptake. As a result, the total amount of AMX sodium may be greater upon application of MN patches to the end-user.

Imperative to the success of MN patches is the ability of the hydrogel-forming MN arrays to successfully insert into the skin. Testing the insertion capabilities of hydrogel-forming MN arrays during stability testing is thus fundamentally important. This study demonstrated that all hydrogel-forming MN arrays packaged in Protect™ 470 foil were capable of inserting into at least three layers of Parafilm M^®^, which is consistent with results from previous studies [33]. However, this was not the case for hydrogel-forming MN arrays packaged in poly(ester) foil or unpackaged MN arrays. Comparing all cohorts, these results provide convincing evidence that MN patches packaged in Protect™ 470 foil could potentially remain hard enough to insert into the skin of the end-user following storage, transport and distribution of these MN patches to hot and humid countries. The results for hydrogel-forming MN arrays unpackaged were not surprising because of the ability of hydrogels, by definition, to absorb large quantities of water and retain this within its structures [50, 51]. Specifically, the moisture absorption properties of citric acid/PVA hydrogels are documented in the literature [52]. In this case, it is reasonable to suggest that the moisture regain is due to the presence of remaining hydroxyl and carboxyl groups of citric acid within the complex structure of the formed three-dimensional hydrogel, hydrogen bonding with water molecules in the moisture. With regard to hydrogel-forming MN arrays packaged in poly(ester) foil, it is reasonable to suggest from the results at this juncture, that this foil failed to fulfil its moisture resistant function during this study, specifically for this MN patch. As a result, this study has demonstrated that the functionality of hydrogel-forming MN arrays is affected when exposed to high humidity. It is recognised that a desiccant is frequently included in primary packaging in order to maintain low RH inside the package [53]. However, this work did not include a desiccant as the goal was to keep the cost of the primary packaging to a minimum due to the end-user being in low resource settings. Height reduction analysis following insertion into Parafilm M^®^ was used to give an indication of the structural functionality of the needles.

AMX sodium DCTs were physically characterised. As previously discussed, physical instability is associated with the change in the appearance of a drug product, but physical instability also encompasses any change of a drug products performance, e.g. hardness and fracturability. It was very encouraging to note that AMX sodium DCTs packaged in Protect™ 470 foil maintained uniform mass, physical dimensions, hardness and fracturability throughout the study period. On the other hand, AMX sodium DCTs packaged in poly(ester) foil significantly distorted in shape, increased in mass, hardness and fracturability. These results were unsurprising following evident discolouration upon visible examination. The change in these physical characterisation parameters could be due to high moisture absorption under high humidity [49]. However, previous studies have reported a decrease in the hardness of tablets following storage at 75% RH rather than an increase as shown in this study [23, 54]. As previously discussed, the degradation of AMX is complex, in that many degradation pathways and degradation products can be formed [41, 42]. With this in mind, one logical reason for increased hardness and fracturability in this work could be due to formation of various AMX degradation products. As the void volume in the AMX sodium DCTs reduced as the amount of degradation products increased as a function of time, the testing parameters, hardness and fracturability both subsequently increased throughout the study period. As previously discussed, poor quality pharmaceutical products are a major public health problem, particularly in hot and humid countries due to improper packaging. Determining the percentage of AMX sodium remaining from AMX sodium DCTs during stability testing is thus fundamentally important. Packaged in Protect™ 470 foil, after 168 days, the percentage of AMX sodium recovered was insignificantly different to the percentage of AMX sodium initially at day 0. However, packaged in poly(ester) foil, a considerable reduction of AMX sodium occurred from day 7 onwards. This considerable reduction of AMX sodium, packaged in poly(ester) foil, is most likely due to the degradation of AMX sodium in the drug-containing reservoir due to aqueous hydrolysis by the imbibed moisture.

Comparing both primary packaging investigated in this study, the results are very encouraging for MN patches packaged in Protect™ 470 foil in terms of hydrogel-forming MN array functionality, AMX sodium DCT physical characteristics and AMX sodium recovery. Thus, Protect™ 470 foil has been identified as a potentially suitable primary packaging to house MN patches containing AMX sodium that could be used for storage, transport and distribution of these MN patches to hot and humid countries. In accordance with the manufacturers specification, Protect™ 470 foil consists of four materials, beginning with the outer material/layer, namely orientated polypropylene, polyethylene, foil and polyethylene, which have all been laminated together to produce this primary packaging [55]. The inner material, polyethylene, melts which allows for this foil to be heat-sealed and the foil provides the moisture barrier properties for this primary packaging. On the other hand, poly(ester) foil, prepared from non-woven poly(ester), failed to provide the moisture barrier properties required for this MN patch containing AMX sodium during stability testing under accelerated storage conditions. As a result, moisture ingress occurred which subsequently affected the AMX sodium MN patches. This preliminary study therefore provides convincing evidence as to the use and importance of a suitable primary packaging for a MN patch to remain ‘fit for purpose’ when it reaches the end-user, specifically hot and humid countries in this case. In accordance with guidelines, one purpose of stability testing is to establish a shelf life for the pharmaceutical product [24, 25]. Product specification for a MN product is currently unknown. In general, the shelf life of a pharmaceutical product is that 90% or more of the drug molecule must remain. Comparing both primary packaging investigated in this study, the results are very encouraging for MN patches packaged in Protect™ 470 foil as the percentage of AMX sodium did not fall below 10% of the initial amount of AMX sodium after being stored for 168 days under accelerated storage conditions.

In accordance to guidelines, another purpose of stability testing is to determine proper storage conditions and suggest labelling instructions for pharmaceutical products [24, 25]. From this preliminary study, these results provide an indication that MN patches packaged in Protect™ 470 foil do not require specific requirements, such as the cold chain during storage, transport or distribution. This finding is a considerable financial benefit. While it is acknowledged that secondary packaging provides all labelling details rather than primary packaging, the determination of recommended labelling statements is important for future studies. From this work, labelling statements are recommended, in accordance with WHO guidelines [24]. As the stability of MN patches under accelerated storage conditions was demonstrated, the first recommended labelling statement on the MN patch packaging would be ‘Do not store above 30 °C.’ Additional labelling statements include ‘Protect from moisture’ and ‘Store in dry condition.’ This is because the MN patches described in this work are hygroscopic in nature.

As MN arrays are a novel delivery system, consideration must be given to, in this case, the health care professionals (HCPs) who will be prescribing, dispensing and in some cases, applying the MN patch. With this in mind, moving forward, future work will involve qualitative studies to assess hydrogel-forming MN patch usability. Feedback will be obtained through in-depth interviews, focus groups or targeted surveys. It is anticipated that usability of prototypes will be assessed with factors such as appropriate application pressure, body site and a need for a feedback indicator considered to confirm successful use.

## Conclusion

As MN arrays progress towards commercialisation, it is important to consider the end-user. Primary packaging is imperative in maintaining the efficiency and stability of labile medicines and MN patches. For the first time, this study investigated the integrity of MN patches containing AMX sodium in different primary packaging when stored under accelerated storage conditions over a test period of 168 days. This study was conducted according to globally accepted standards, the ICH and WHO guidelines. At pre-defined intervals during stability testing, the performance of the MN patch was assessed, in terms of hydrogel-forming MN array functionality, AMX sodium DCT physical characteristics and AMX sodium recovery. Comparing both primary packaging, this study successfully demonstrates that Protect™ 470 foil is more effective than poly(ester) foil in terms of moisture barrier function and temperature resistance for these MN patches. Therefore, this primary packaging has been identified as a potentially suitable primary packaging that could be used for storage, transport and distribution of MN patches containing AMX sodium to hot and humid countries. This work indicates the importance of investigating the stability of MN patches in primary packaging, intended for MN-mediated transdermal delivery so that they are ‘fit for purpose’ when they reach the end-user. The presentation of this evidence also provides a foundation for further research in this area including qualitative studies to assess MN patch usability.
